# More than a headache—somatic and mental symptom burden in spontaneous intracranial hypotension before and after surgical treatment

**DOI:** 10.3389/fneur.2024.1421579

**Published:** 2024-10-08

**Authors:** Florian Volz, Claas Lahmann, Katharina Wolf, Christian Fung, Mukesch Johannes Shah, Niklas Lützen, Horst Urbach, Charlotte Zander, Jürgen Beck, Amir El Rahal

**Affiliations:** ^1^Department of Neurosurgery, Medical Center – University of Freiburg, Freiburg, Germany; ^2^Department of Psychosomatic Medicine and Psychotherapy – Center for Mental Health, Medical Center – University of Freiburg, Freiburg, Germany; ^3^Department of Neurosurgery, Lindenhofspital, Bern, Switzerland; ^4^Department of Neuroradiology, Medical Center – University of Freiburg, Freiburg, Germany; ^5^Faculty of Medicine, University of Geneva, Geneva, Switzerland

**Keywords:** spontaneous intracranial hypotension, spinal CSF leak, clinical presentation, headache, depression

## Abstract

**Introduction:**

Patients with spontaneous intracranial hypotension (SIH) frequently present with several symptoms and impaired mental health. This study systematically investigates the somatic and mental symptom burden of SIH and the effect of surgical treatment.

**Methods:**

In this single-center retrospective study, patients with surgical closure of a spinal cerebrospinal fluid leak between September 2022 and July 2023 completed the Somatic Symptom Scale (SSS-8), the Somatic Symptom Disorder – B Criteria Scale (SSD-12), and the Patient Health Questionnaire (PHQ-8) preoperatively and three and 6 months postoperatively.

**Results:**

Fifty-seven patients were included. All three scores showed clearly pathological values before surgery (SSS-8: 12 [IQR 6.5–16], SSD-12: 26 [IQR 19.5–33.5], PHQ-8: 11 [IQR 6.5–15]) representing a high somatic symptom burden and relevant current depression. After surgery, there was a significant and sustainable improvement (SSS-8: 8 [IQR 3–11.75], SSD-12: 12.5 [IQR 5–21.75], PHQ-8: 4.5 [IQR 2–9], *p* < 0.001, respectively) that exceeded the minimal clinically important difference for every score.

**Conclusion:**

SIH presents with high somatic and mental symptom burden. Surgical treatment leads to a relevant improvement of somatic and depressive symptoms. However, even after surgical success some patients still exhibit elevated depressive scores. Depressive symptoms might be added to the typical symptomatology of SIH.

## Introduction

Spontaneous intracranial hypotension (SIH) can present with a wide variety of symptoms, with acute orthostatic headache often being the most prominent (1–4). Symptoms are usually assessed based on free self-reported information from the patients without systematic evaluation using validated scores. In addition to headache, dizziness, nausea, tinnitus, and cognitive symptoms (“brain fog”) are frequently reported debilitating symptoms. A severe, albeit rather rare, clinical manifestation of SIH is progressive behavioral and cognitive impairment due to severe brain sagging with downwards herniation of the brainstem and the cerebellum (5–8). The combination of these different symptoms leads to a significant reduction in quality of life (9, 10). In many cases, SIH patients are younger, previously healthy individuals with minimal comorbidity (9) who find their daily lives completely disrupted by the condition. It is, therefore, highly likely that the somatic burden is accompanied by psychological and mental distress. While this is cursorily mentioned in a few publications (3), there is only little systematic data available, especially on the time course after surgical treatment of SIH (11).

Microsurgical closure of the responsible spinal cerebrospinal fluid (CSF) leak is a viable treatment option in cases of unsuccessful conservative treatment (2). Most studies about the treatment effect focus on headache, quality of life or a general change (9, 12–15). To date, there has been no systematic consideration of other, not necessarily classic somatic complaints and psychosomatic symptom perception.

This study aims to systematically assess the somatic symptom burden, the symptom perception, and the depressive symptom severity in a large representative cohort of patients before and after microsurgical closure of a spontaneous spinal CSF leak.

## Methods

This single-center retrospective study from a highly specialized tertiary center used systematically collected “Patient-reported outcome measures” (PROMs) in patients with surgical sealing of spontaneous spinal CSF leaks between September 2022 and July 2023. The study was approved by the local Ethics Committee (22-1512-S1-retro), and the patients gave their informed consent for the evaluation of the PROM data. The study followed the STROBE guidelines (16). Inclusion criteria were (i) SIH, according to the 3rd edition of the International Classification of Headache Disorders (17), and (ii) confirmation and surgical treatment of a spinal CSF leak. Exclusion criteria were (i) no reliable contact possibility for the follow-up, (ii) insufficient language skills to complete the questionnaires in English or German, and (iii) no consent.

### Patient-reported outcome measures

#### Somatic Symptom Scale–8 (SSS-8)

The Somatic Symptom Scale–8 (SSS-8) assesses the somatic symptom burden by rating eight common somatic complaints (18), including headache, dizziness and fatigue. Each of the eight items is scored from 0 to 4 points, resulting in a summary score from 0 to 32. Suggested cut-off scores are <4 for minimal, 4–7 for low, 8–11 for moderate, 12–15 for high, and 16–32 for very high somatic symptom burden (18). A change of 3 points reflects the minimal clinically important difference (MCID) (19). The item assessing impairment due to headache does not address positional dependency or further differentiate between different types of headaches.

#### Somatic Symptom Disorder – B Criteria Scale (SSD-12)

The Somatic Symptom Disorder – B Criteria Scale (SSD-12) assesses the B criteria (=psychological features) of the somatic symptom disorder (SSD) as defined in the 5th edition of the Diagnostic and Statistical Manual of Mental Disorders (DSM-5) (20). It quantifies the perception of symptom-related feelings (affective aspect), behavior (behavioral aspect), and thoughts (cognitive aspect) as the three main dimensions of the subjective burden of persistent physical complaints (21, 22). Four specific questions represent each subscale (Affect, Behavior, Cognition), each scored from 0 to 4 points, respectively. The sum of all points gives the SSD-12 summary score between 0 and 48 points. A change of 3 or more points is considered the MCID (23).

#### 8-item Patient Health Questionnaire (PHQ-8)

The 8-item Patient Health Questionnaire (PHQ-8) measures depressive symptom severity (24, 25). Each item is scored from 0 to 3 points, resulting in a score of 0 to 24. Suggested cut-offs are 5 for mild, 10 for moderate, 15 for moderate, and 20 for severe depression, respectively (24). Scores of 10 or higher are indicative of relevant current depression (24, 26, 27). Depending on the statistical method, a change of 3–6 points is accepted as MCID (28, 29). In order to avoid possible medico-legal problems associated with an automated follow-up, the PHQ-8 was deliberately used in the current study, as it does not include a question about acute suicidality.

Using a digital PROM platform with an automated follow-up, the questionnaires were completed after confirmation of the type and location of the CSF leak and after informed consent for the surgical treatment (baseline) as well as three and 6 months after surgery. Clinical and diagnostic parameters like sex, age, weight, height, Body Mass Index (BMI), symptom duration before surgery and type of spinal CSF leak were analyzed retrospectively after systematic collection in the internal database. Comorbidity before surgery was assessed via the 13-item Self-administered Comorbidity Questionnaire (SCQ), with particular regard if “depression” was stated as a relevant medical condition. Complications, namely a new neurological deficit after surgery and/or the need for revision surgery, were retrospectively analyzed via the routine postoperative follow-up consultation with the patients.

#### Surgical treatment

A unilateral, minimally invasive tubular approach was used in all cases (30). Ventral leaks (Type 1) were closed via the microsurgical intradural/extradural sandwich patching technique (31), lateral leaks (Type 2) via microsurgical fibrin-patch augmentation with or without additional clipping or suturing, and CSF-venous fistulas (Type 3) via microsurgical thermocoagulation of the draining vein and clipping of the nerve root. Initial confirmation of leak closure relied on the intraoperative judgment of the two surgeons that performed all cases (JB or FV) and the clinical improvement in the first week post-surgery. Final confirmation of successful closure was established through clinical assessment and a routine MRI of the brain and spine conducted 3 months after surgery. During the hospitalization period, no patient had a psychiatric or psychotherapeutic evaluation or intervention, and no modifications to preexisting psychiatric medications were made.

### Statistics

Statistical analysis was performed using SPSS Statistics (IBM, Version 29.0). As all preoperative PROM scores showed a normal distribution and the postoperative scores a skewed distribution, all results were presented as median with interquartile range (IQR) for better readability. The Wilcoxon signed-rank test was used to compare the baseline and the three-and six-month follow-up scores, respectively. In the SSS-8, the influence of each of the eight different items on the summary score was analyzed separately. For the item specifically addressing headache, the distribution of the 5 possible answers was additionally evaluated for each time point. Subgroup analyses with the Mann–Whitney-U-Test were performed for a symptom duration of <90 or ≥ 90 days as proposed in previous series (9, 12) in all scores and for patients with preoperative PHQ-8 scores of <10 or ≥ 10.in the SSS-8 and SSD-12. *p*-values <0.05 were considered statistically significant.

## Results

### Study population

**Figure 1:**
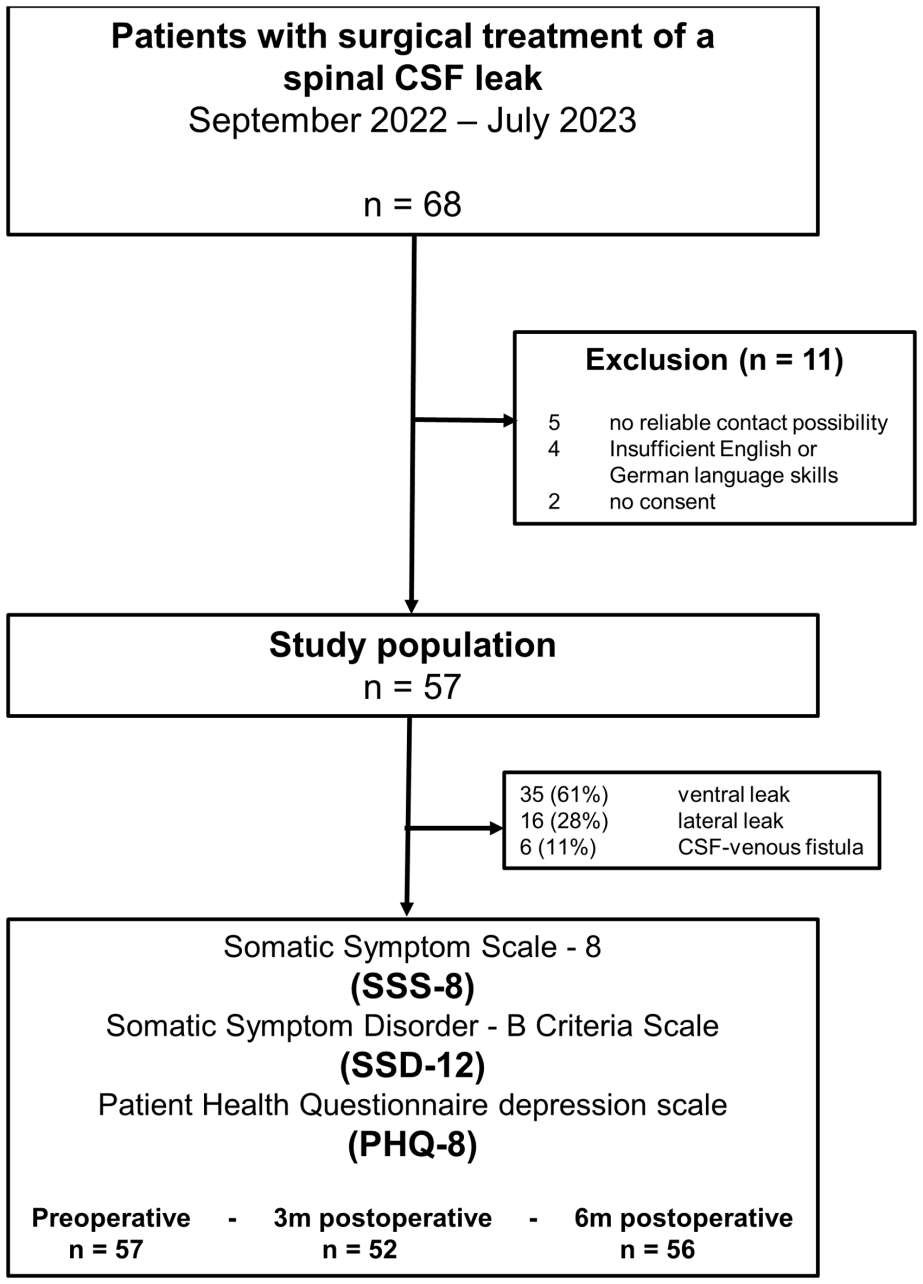
Flowchart displaying the selection of the patients.

Since the regular implementation of the SSS-8, SSD-12, and PHQ-8 questionnaires in the electronic PROM system, 68 patients had surgery for a spontaneous spinal CSF leak between September 2022 and July 2023. Eleven patients were excluded from the analysis: five patients (all with CSF-venous fistulas and older than 75 years) because of no reliable email contact for the automated follow-up; four patients because of insufficient German or English language skills to complete the questionnaires; two patients declined to complete the questionnaires. Thus, the study population consisted of 57 patients (Figure 1). Most patients (46/57, 81%) lived in Germany at the time of surgery, with the rest residing in different Western European countries. The follow-up was nearly complete, with a 3-month follow-up of 91% (52/56) and a 6-month follow-up of 98% (56/57), respectively.

The majority of surgeries were for ventral leaks (35/57, 61%), followed by lateral leaks (16/57, 28%) and CSF-venous fistulas (6/57, 11%). The median age was 45 years (IQR 37–56; range 24–71 years), and 32/57 (56%) were female (Table 1). Symptom duration before the surgery was 177 days (IQR 113–427; range 23 days – 15 years). Ten patients (17.5%) had a symptom duration of less than 90 days, 45 patients (79%) of 90 days or longer, and two patients (3.5%) had no rememberable onset of specific symptoms. Before the index surgery, all patients had undergone at least 3 weeks of strictly conservative treatment including bed rest, fluid substitution, and/or caffeine intake either in another institution or as outpatients. Thirty of 57 patients (53%) had one or several epidural epidural blood patches without complete resolution of symptoms and one patient had an unsuccessful surgical procedure in another center. The preoperative comorbidity was generally low, with a median SCQ summary score of 2 (IQR 0–4). Eight of 57 patients (14%) reported depression as a comorbidity, and 5/57 (9%) reported a depression-related treatment. Chronic subdural hematoma was present in 15/57 patients (26%), with mostly bilateral expression (13/57, 22%). Three patients (two unilateral, and one bilateral) needed operative treatment for the hematoma via twist drill craniostomy before the spinal surgery due to a relevant mass effect.

**Table 1:** Patient characteristics.

Patient characteristics	*N* = 57	
Female/Male	32/25	56%/44%
	Median (IQR)	Range
Age (years)	45 (37–56)	24–71
Body Mass Index (BMI, kg/m^2^)	24.5 (21.7–28.0)	16.9–33.8
Weight (kg)	75 (63–89)	50–112
Height (m)	1.73 (1.68–1.79)	1.53–1.94
Comorbidity		
	Median (IQR)	Range
SCQ Summary score	2 (0–4)	0–9
Self-reported depression	8	14%
Depression-related treatment	5	9%
Duration of symptoms		
	Median (IQR)	Range
Days	177 (113–427)	23–5644
Duration ≤90 days	10	18%
Duration >90 days	45	79%
No clear symptom onset	2	3%
Type of spinal CSF leak		
Ventral leak (Type 1)	35	61%
Lateral leak (Type 2)	16	28%
CSF-venous fistula (Type 3)	6	11%
	Median (IQR)	Range
Length of stay after surgery (days)	4 (4–5)	3–7

CSF, Cerebrospinal Fluid; SCQ, Self-administered Comorbidity Questionnaire.

### Patient-reported outcome

All patient-reported outcomes (SSS-8, SSD-12 summary score, and PHQ-8) showed consistent results, significantly improving from preoperatively to 3 and 6 months after surgery, respectively. The discrete alterations between 3 and 6 months were not statistically significant (Table 2).

**Table 2:** Patient-reported outcome measures preoperative and 3 and 6 months postoperative.

		Preoperative	3 months postoperative	6 months postoperative
		*N* = 57	*N* = 52	*N* = 56
		Median (IQR)	Median (IQR)	Median (IQR)
Somatic Symptom Scale–8 (SSS-8)		12 (6.5–16)	8 (4–14)	8 (3–11.75)
Preoperative PHQ-8 < 10 points	*N* = 21/57 (36.8%)	5 (4–8)	5 (2–11)	4.5 (1–7.25)
Preoperative PHQ-8 ≥ 10 points	*N* = 36/57 (63.2%)	14.5 (11–18.25)	9 (5.5–16)	9.5 (6.75–15.5)
Somatic Symptom Disorder – B Criteria Scale (SSD-12) Summary Scale		26 (19.5–33.5)	16.5 (6–26.75)	12.5 (5–21.75)
Preoperative PHQ-8 < 10 points	*N* = 21/57 (36.8%)	20 (16–26)	13 (6–22)	10 (3.75–17)
Preoperative PHQ-8 ≥ 10 points	*N* = 36/57 (63.2%)	30 (23–36)	19 (6.5–32)	15.5 (9.75–24.25)
SSD-12 Affective Subscale		9 (7–12)	7 (2.25–11.75)	6 (3–8.75)
SSD-12 Behavior Subscale		9 (5.5–12.5)	4.5 (1–9)	4 (0.25–7)
SSD-12 Cognition Subscale		8 (6–10)	4 (2–8)	4 (2–7)
Patient Health Questionnaire (PHQ-8)		11 (6.5–15)	6 (3–9)	4.5 (2–9)

SSS-8, Somatic Symptom Scale–8; SSD-12, Somatic Symptom Disorder – B Criteria Scale; PHQ-8, Patient Health Questionnaire.

### Somatic Symptom Scale–8 (SSS-8)

**Figure 2:**
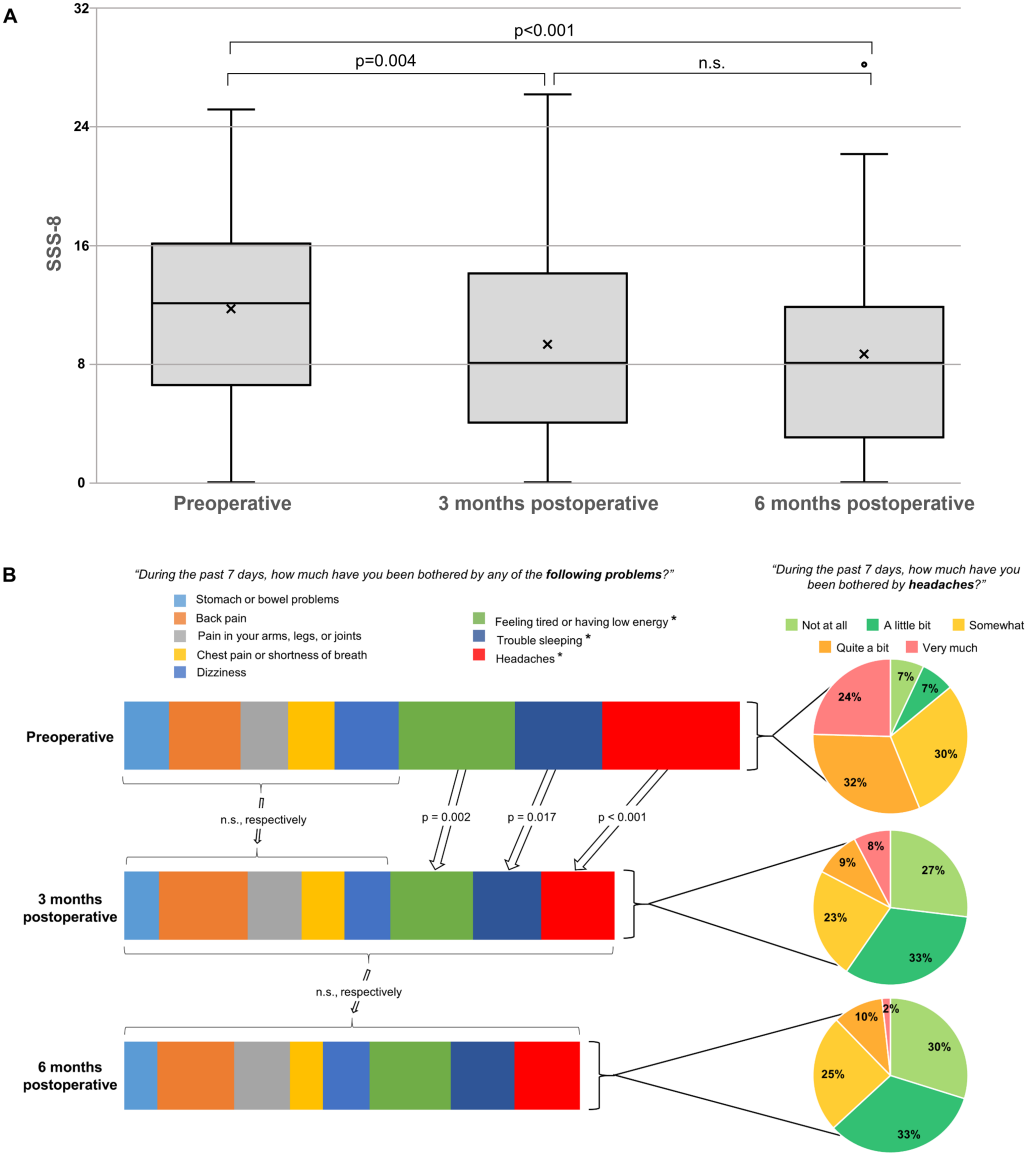
**(A)** Boxplots representing the Somatic Symptom Scale – 8 (SSS-8) scores. The line in the box represents the median, and the x represents the mean. The median preoperative SSS-8 score of 12 indicates a high somatic symptom burden. Postoperatively, there is a significant improvement at 3 months (*p* = 0.004) and a further at 6 months (*p* < 0.001), without significant difference (n.s.) after 3 months. **(B)** Bar graphs on the left representing the eight different somatic complaints of the SSS-8: 3 months postoperatively, the item “headache” shows the most prominent improvement, followed by “feeling tired or having low energy” and “trouble sleeping.” The other five complaints remain nearly unchanged (n.s.). The pie charts on the right represent the separate evaluation of the item “headache” and show a significant reduction in the rate of headache with a high and very high impact.

The preoperative SSS-8 summary score of 12 (IQR 6.5–16) significantly improved to 8 (IQR 4–14, *p* = 0.004) at the 3-month follow-up and to 8 (IQR 3–11.75, *p* < 0.001) at the 6-months follow-up (Figure 2). The overall improvement of the SSS-8 summary score was mainly caused by the reduction of the impairment due to headache. The “headache item” showed the most prominent improvement, followed by problems of “feeling tired or having low energy” and having “trouble sleeping.” Only these three items experienced a significant change, whereas the other five complaints remained nearly unchanged (Figure 2B). The separate evaluation of the “headache item” showed a significant reduction in the rate of headache with a high and very high impact (Figure 2B).

### Somatic Symptom Disorder – B Criteria Scale (SSD-12)

**Figure 3:**
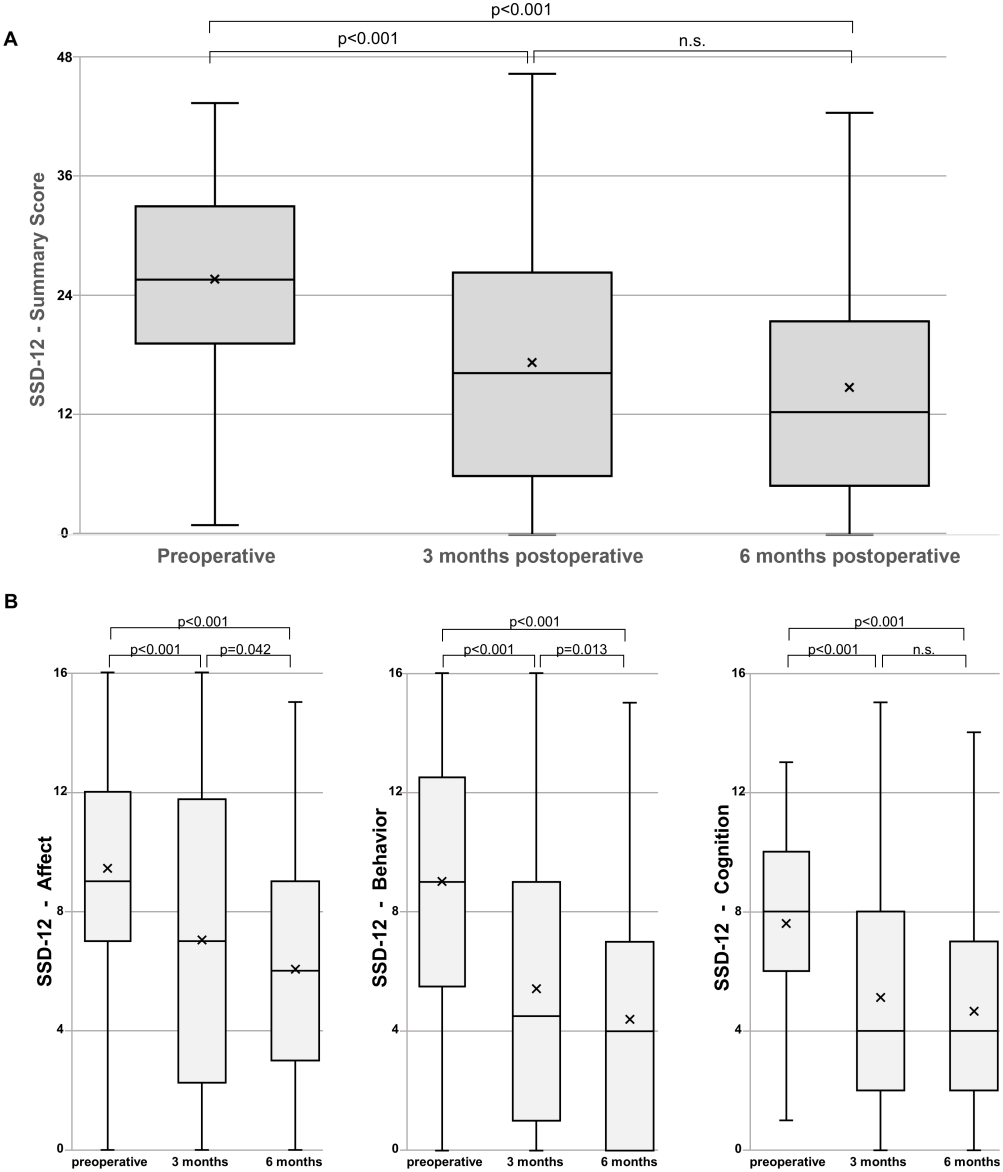
**(A)** Boxplots representing the Somatic Symptom Disorder – B Criteria Scale (SSD-12) summary scores. The line in the box represents the median, and the x represents the mean. The median preoperative SSD-12 summary score of 26 significantly improves to 16.5 at 3 months (*p* < 0.001) and a further 12.5 at 6 months (*p* < 0.001) without significant difference (n.s.) between the follow-up dates. **(B)** Boxplots representing the SSD-12 Affect, Behavior, and Cognition subscales. All three sub-criteria significantly improve with the affective and behavioral sub-criteria further improving significantly between three and 6 months (*p* = 0.042 for affect and *p* = 0.013 for behavior).

From a preoperative SSD-12 summary score of 26 (IQR 19.5–33.5), there was a significant improvement to 16.5 (IQR 6–26.75, *p* < 0.001) at the 3-months and further to 12.5 (IQR 5–21.75, *p* < 0.001) at the 6-months follow-up (Figure 3). The further improvement in the summary score between three and 6 months was not statistically significant (*p* = 0.075). Notably, all three sub-criteria for affective, behavioral, and cognitive dimensions significantly improved. The affective and behavioral sub-criteria continued to improve significantly between three and 6 months (*p* = 0.042 for affect and *p* = 0.013 for behavior), whereas the cognitive sub-criterion did not further improve significantly after 3 months (*p* = 0.374).

### 8-item Patient Health Questionnaire (PHQ-8)

**Figure 4:**
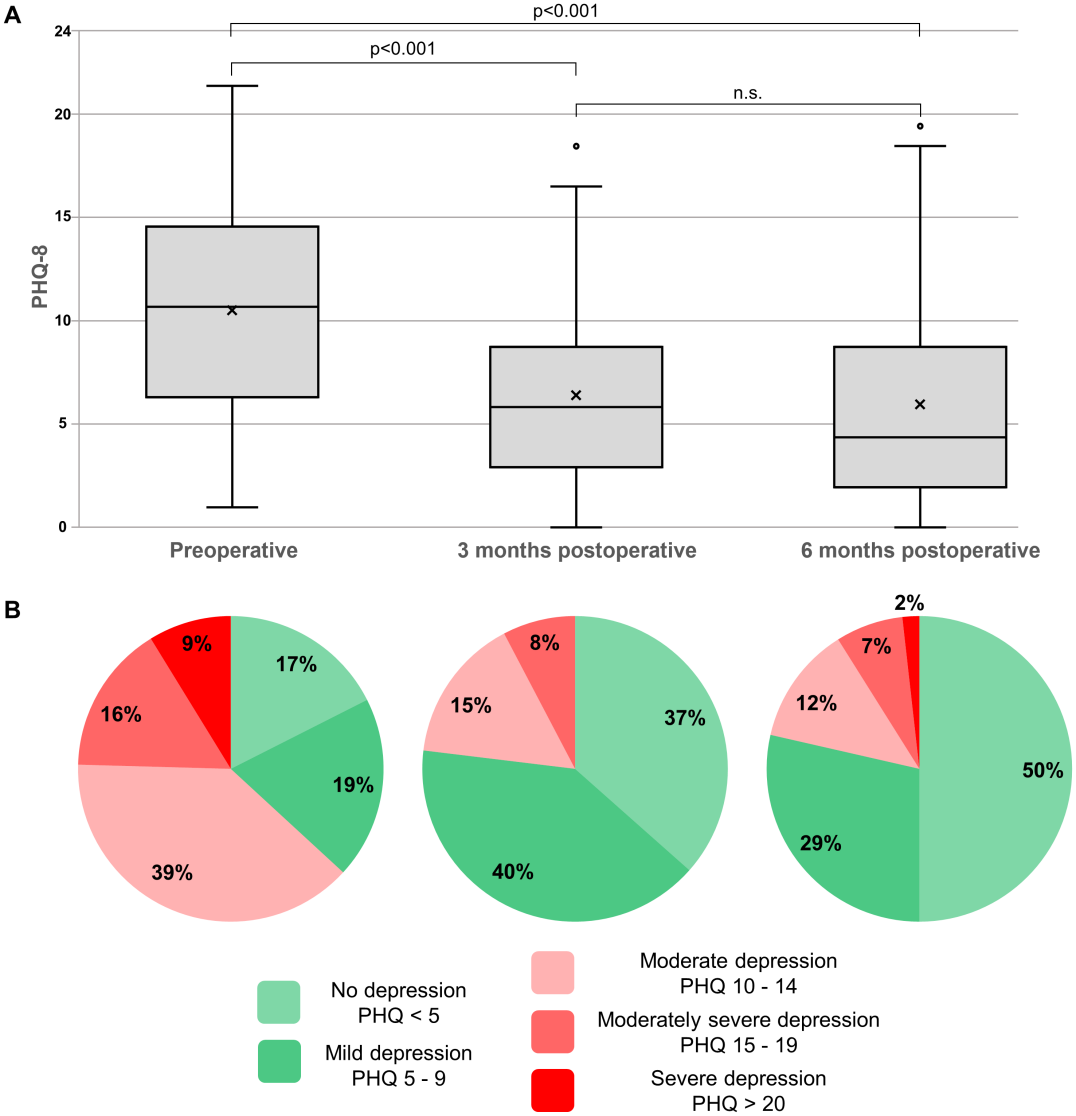
**(A)** Boxplots representing the 8-item Patient Health Questionnaire (PHQ-8). The line in the box represents the median, and the x represents the mean. Scores of 10 or higher are indicative of relevant current depression. The median preoperative PHQ-8 score of 11 significantly improves to 6 at 3 months (*p* < 0.001) and a further 4.5 at 6 months (*p* < 0.001) without significant difference (n.s.) after 3 months. **(B)** Pie charts representing the percentage of patients with relevant depressive symptoms (PHQ-8 scores ≥10, red colors) according to the suggested cut-off values. Preoperatively, ≈64% of patients have a PHQ-8 score of ≥10; 6 months after surgery, only ≈21%.

The preoperative PHQ-8 score of 11 (IQR 6.5–15) significantly decreased to 6 (IQR 3–9, *p* < 0.001) at the 3-month follow-up and to 4.5 (IQR 2–9, *p* < 0.001) at the 6-months follow-up (Figure 4). Preoperatively, 63.2% of patients (36/57) had a PHQ-8 score of ≥10, suggestive of relevant depression. Three months after surgery only 23.1% of patients (12/52) had a PHQ of ≥10, and 6 months after surgery only 21.4% (12/56) (Figure 4B).

### Subgroup analysis

Patients with preoperative PHQ-8 scores <10 (36.8%) showed consistently lower values in both the SSS-8 and the SSD-12 than patients with PHQ-8 scores of 10 and higher (63.2%) at all time points (Table 2). Notably, in the SSS-8, there was a significant difference at baseline (5 vs. 14.5, *p* < 0.001) and at both the 3-month (5 vs. 9, *p* = 0.038) and 6-month (4.5 vs. 9.5, *p* = 0.001) follow-up. In the SSD-12, the only statistically significant difference was at baseline (20 vs. 30, *p* = 0.001) but not at the 3 months (13 vs. 19, *p* = 0.418) and 6 months (10 vs. 15.5, *p* = 0.091) follow-up.

Subgroup analysis for symptom duration revealed slightly higher median values in all scores (SSS-8, SSD-12, and PHQ-8) preoperatively for a symptom duration >90 days but no significant difference: (SSS-8: 9.5 vs. 13, *p* = 0.364; SSD-12: 22.5 vs. 27, *p* = 0.143; PHQ-8: 10.5 vs. 11, *p* = 0.751). Notably, there was a significant difference at the 3-month follow-up for all scores, with favorable outcomes for the shorter symptom duration: (SSS-8: 5.5 vs. 10, *p* = 0.017; SSD-12: 5 vs. 20, *p* = 0.006; PHQ-8: 3.5 vs. 7, *p* = 0.039). At the 6-month follow-up, both subgroups had reached a similar outcome with a significant difference only in the SSD-12 (SSS-8: 7 vs. 8, *p* = 0.156; SSD-12: 10 vs. 14, *p* = 0.02; PHQ-8: 3 vs. 5, *p* = 0.118).

### Surgical complications

Three patients (all ventral leaks) needed revision surgery: one due to insufficient visualization of the ventral leak in the primary surgery (“wrong level surgery”) after 4 days; one due to insufficient closure of the dorsal durotomy after 10 days; one because of an epidural hematoseroma after 14 days. These patients were included in the analysis only once, with the baseline values from before the first surgery and postoperative values corresponding to the date of the second surgery, respectively. There was no persistent motor deficit after surgery. In the cases where a thoracic nerve root was clipped, there was an expectable unilateral hypesthesia in the corresponding dermatome, without impact on the patient’s recovery or the need for a specific treatment. Length of stay after surgery was 4 days (IQR 4–5; range 3–7 days), No patient needed an intervention for chronic subdural hematoma after surgery for the spinal CSF leak.

## Discussion

This study systematically examines various somatic complaints (via the SSS-8), individual psychological symptom perception (via the SSD-12), and depressive symptoms (via the PHQ-8) before and after neurosurgical sealing of a spinal CSF leak with a 6-month follow-up. All scores show a significant and sustainable improvement. The significant reduction in somatic symptom burden is mainly due to a reduction in headache impairment and, to a smaller degree, to a decrease in perceived fatigue and sleep disturbances. The study confirms the high impact of spinal CSF leaks on mental health, with a high rate of relevant depressive symptoms preoperatively and a significant reduction postoperatively.

### Somatic symptom burden

Patients with SIH often present with a multifaceted symptom complex that frequently goes beyond the classic orthostatic headache (1, 2). Typically, these symptoms are collected through free, unstructured patient reporting. Such data are not easily comparable with each other and are not ideal for measuring the effect of a treatment. Using a validated score to estimate the burden of different symptoms allows a more accurate comparison and statistical evaluation of the change after an intervention. Obviously, the eight distinct dimensions of the SSS-8 cannot fully cover the entire spectrum of SIH symptoms. However, “headache,” “dizziness,” and “feeling tired or having low energy” are some of the most commonly reported. Applying the proposed cut-offs (18), the preoperative median SSS-8 score of 12 represents a high somatic symptom burden. The sustainable improvement from 12 to 8 points 6 months after surgery is not only highly statistically significant but also a clinically highly relevant difference (19).

The beneficial effect of surgical closure of spinal CSF leaks on headache has been previously investigated using the Headache-Impact-Test (9, 13). Though the SSS-8 certainly cannot be considered a specific score for assessing headache impact, the separate analysis of the headache item of the SSS-8 confirms the previous findings of high headache impact before and a clinically relevant improvement after surgery. Admittedly, the headache sub-item of the SSS-8 is rather superficial without consideration of frequency, intensity or orthostatic nature of the headache. However, as a more detailed examination of headache focusing on the orthostatic nature in patients with SIH has already been addressed (13), this current study deliberately focuses on a broader spectrum of symptoms without specific characterization of the headache. Comparable to this study (13), the current results also show a relevant proportion of residual headache even after radiological confirmation of a successful closure of the leak (Figure 2B). Possible explanations include pre-existing, non-SIH-related types of headache or postoperative rebound-hypertension, however, more extensive and ideally prospective studies are necessary.

In addition to headache burden, there is a significant improvement in “feeling tired or having low energy,” a frequently reported symptom of SIH under the term “fatigue” (1, 2). In contrast to these two widespread complaints, “sleep disturbance” is less frequently reported. However, we also found a significant improvement (Figure 2B). Notably, we found no significant improvement in the SSS-8 item “dizziness,” a commonly reported symptom of SIH (1, 2). Considering that patients may summarize different complaints under this term, this is consistent with the clinical observation that other otovestibular symptoms, such as hearing impairment and tinnitus, tend to improve less or with more latency after surgery. The unchanged scores for back or arms/legs/joints pain after surgery may be interpreted as further evidence that the minimally invasive approach (30) has a favorable morbidity/benefit ratio. However, a follow-up of 6 months is clearly not enough to reliably assess long-term sequela in terms of spinal degeneration or instability.

### Effect of SIH on mental health

While consistently highlighting the various and sometimes severe somatic symptoms of SIH, most reviews address the impact on mental health not at all or only superficially (1–4). In a recently published series, we reported the high impact of SIH on mental health using the 21-item version of the Depression Anxiety Stress Scales and the significant improvement after surgical treatment (11). The current study used the PHQ-8, a validated instrument for assessing depressive symptoms (24, 32, 33). As a self-administered questionnaire, the PHQ-8 is not suitable for definitively diagnosing depression or for estimating its exact prevalence in a population (34, 35). However, it is an efficient tool for the screening and monitoring of depressive symptoms and for assessing the effect of a medical intervention (24, 32, 33). Elevated levels of depression, measured with the PHQ are well documented in spinal conditions, for example in multiple studies about traumatic spinal cord injury (36).

This study confirms the high impact of SIH on mental health as well as the effectiveness of surgical sealing of the responsible CSF leak. A previously published study demonstrated significantly elevated levels of depression, anxiety, and stress using the Depression Anxiety Stress Scales (DASS-21), The current study focuses on depressive symptoms (via the PHQ-8) and the patient’s focus on physical symptoms (via the SSD-12). The preoperative PHQ-8 score of 11 is above the cut-off score of 10, which indicates relevant depressive symptoms (24, 26, 27). Six months after surgery, the score improved to 4.5 (indicating no relevant depression), with the entire interquartile range (IQR 2–9) reaching scores below 10. This improvement exceeds even the more conservative MCID values, which are accepted to be between 3 and 6 points (28, 29). When applying the suggested cut-offs (24, 26, 27), more than 63% of patients preoperatively show a relevant depressive syndrome (Figure 4B). Even taking into account the above-mentioned limitations, this value is considerably higher than the prevalence of depressive disorders in Germany (approximately 10%) or Europe (approximately 6%) (27, 37). Six months after surgery, the proportion of patients with PHQ-8 scores ≥10 drops significantly to 21%. Although this decrease represents a significant improvement in the depressive symptom burden, it remains clearly above the assumed prevalence levels of relevant depression. Whether this is partly due to a pre-existing depression independent of SIH or partly due to a persistent long-term consequence of the SIH disease cannot be definitively answered based on the available study results. Therefore, we suggest intensifying the research on spinal CSF leaks and depression. The high rate of relevant depressive symptoms in patients with a spinal CSF leak, and the sustainable improvement after surgical treatment, might potentially lead to a condition in terms of a spinal (CSF leak associated) depression. In selected cases, and especially if other SIH typical somatic complaints are present, an additional diagnostic work-up for a spinal CSF leak might be included in a psychiatric depression assessment.

Unfavorable, higher PHQ-8 scores were associated with an increased somatic symptom burden, indicated by higher SSS-8 scores (Table 2). Initially, patients with PHQ-8 scores ≥10 showed a high somatic symptom burden, which reduced to moderate levels at the 6-month follow-up, demonstrating significant improvement. Conversely, patients with lower PHQ-8 scores (<10) maintained a low somatic symptom burden throughout the study. The main improvement in somatic symptoms was observed in the group with initially high PHQ-8 scores. The Somatic Symptom Disorder (SSD) is characterized by an inappropriate and unusually high level of emotions (e.g., anxious feelings as an affective criterion), actions (e.g., disproportionate amount of time or energy spent dealing with symptoms as a behavioral criterion), and excessive thoughts (e.g., perseverating on the seriousness of symptoms as a cognitive criterion), in dealing with somatic symptoms (20–22). The somatic symptoms may or may not be caused by another medical condition. The patients in the current study show a median baseline SSD-12 summary score of 26, which is significantly higher than in the general German population (mean 7.85, SD 9.3 (22)). The improvement after surgery (to 16.5 at 3 months and to 12.5 at 6 months) is clearly above the 3 points that are considered clinically relevant (23) (Table 2 and Figure 3). However, the values remain higher than in the general population. Notably, the SSD-12 is the only score in this study where significantly worse scores persisted from baseline up to the 6-month follow-up when the symptom duration was greater than 90 days.

A marked change in behavior, impetus, and cognitive function is described as a sequelae of severe midbrain deformation and brain sagging in SIH (5–8). The patients present with a clinical syndrome of a behavioral-variant frontotemporal dementia (bvFTD). Albeit the fact, that bvFTD is a very rare yet severe complication of SIH and as the current cohort did not comprise such severe brain sagging deformation as described in these cases, it could be hypothesized that the changes observed in affective, behavioral, and cognitive dimensions of the SSD-12 or the depressive symptoms measured via the PHQ-8 reflect a similar pathomechanism.

Our results, with substantially elevated baseline scores on the PHQ-8 and SSD-12, emphasize the severe impact of SIH on mental health. While there is a noticeable improvement, both scores are still elevated at 6 months, highlighting the long-term effect of the condition on mental health even after surgical success. A broader and systematic use of appropriate scores in the assessment of SIH patients seems reasonable, to highlight the condition’s impact beyond purely somatic complaints as well as to monitor long-term sequelae. As SIH and spontaneous spinal CSF leaks might still be an underdiagnosed condition, the inclusion of psychological symptoms as an additional complaint seems advisable. Additionally, it might be worth exploring the possibility that spinal leaks could contribute to depression or depressive symptoms, potentially leading to a condition we might refer to as spinal (CSF leak associated) depression.

### Limitations

This study has several limitations. Although all outcome measures were systematically collected and the follow-up is almost complete, it is a retrospective single-center series with a highly selected patient cohort. The mandatory inclusion criterion of surgical treatment only allows a statement about this specific patient cohort without general applicability to all patients with SIH and different treatment strategies. The design of the SSS-8 with eight predefined somatic complaints does not allow the inclusion of other symptoms or a specific characterization of the headache. However, it permits a systematic and comparable evaluation of some of the most frequent symptoms. Obviously, self-report questionnaires do not replace expert assessment, e.g., structured interviews or validated external rating scales, and are therefore not suitable for confirming any psychiatric or headache diagnosis. However, it is a very efficient way to include psychological parameters in the clinical assessment and to monitor changes in symptom burden after surgical treatment.

## Conclusion

Spontaneous intracranial hypotension often presents with a high somatic symptom burden and a severe impact on mental health. Surgical closure of the responsible CSF leak leads to a relevant reduction of somatic symptoms, significantly improving headache, fatigue and sleeping problems. Depressive symptoms are also significantly reduced after surgery. However, even after surgical success, a considerable number of patients still exhibit elevated depressive symptom scores. Because of their high frequency and impact, psychological symptoms like depression should be included in the symptomatology of SIH. In selected cases, with typical somatic complaints, a depression assessment might include an additional work-up for a spinal CSF leak.

## Data Availability

The raw data supporting the conclusions of this article will be made available by the authors, without undue reservation.
